# Supplementary figures and images for: Generalization of Classification of AlkB Family Alkane Monooxygenases from Rhodococcus (sensu lato) Group Based on Phylogenetic Analysis and Genomic Context Comparison
Source: Int J Mol Sci. 2025 Feb 17;26(4):1713. doi: 10.3390/ijms26041713 (PMC11854999; doi:10.3390/ijms26041713)

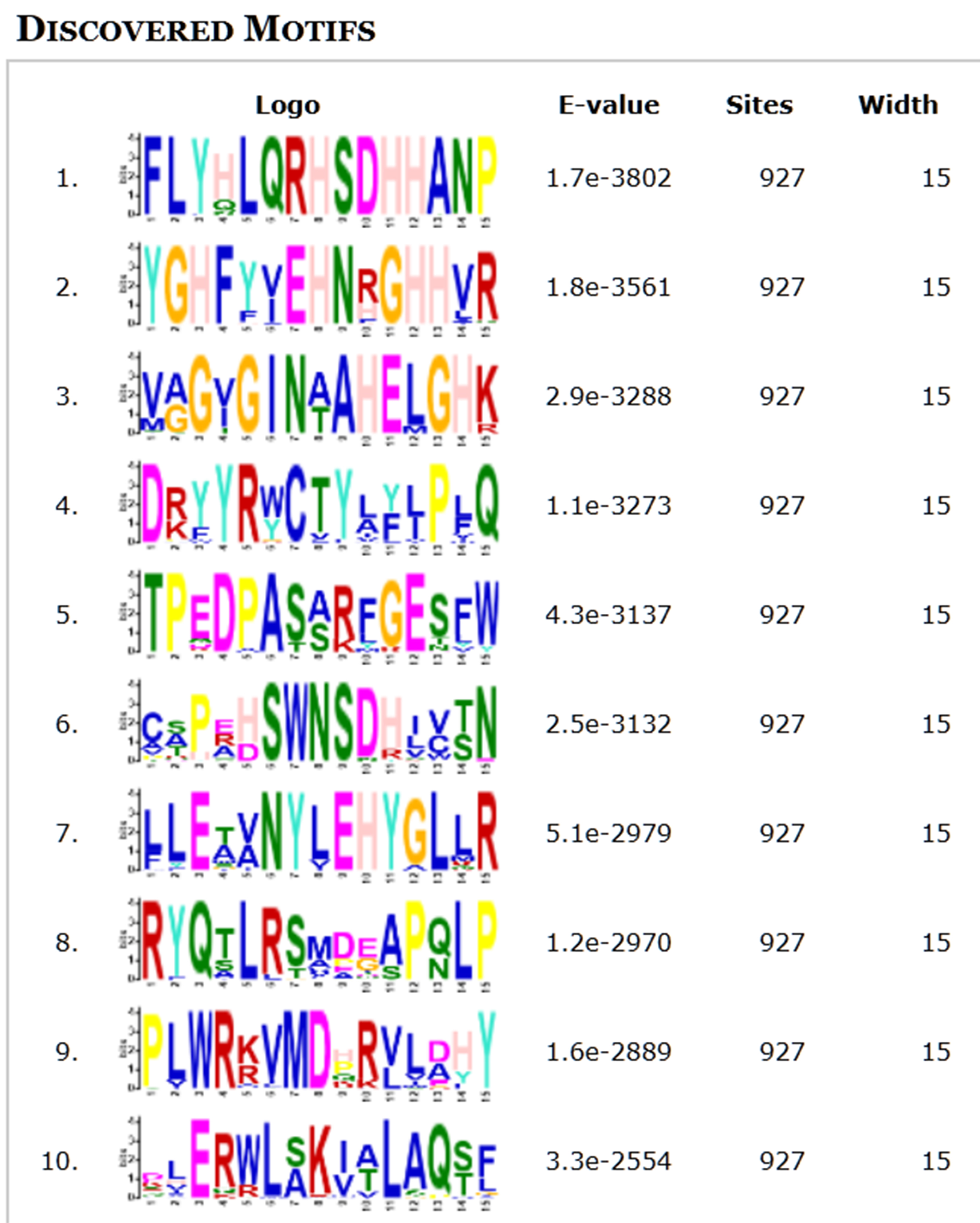

Supplement: Supplementary file 1 [file ijms-26-01713-s001.zip › Petrikov_et_al_Supplementary Figure S1 Motifs discovered by MEME tool in AlkB protens.jpg]
